# Supplementary material for: Complex‐centric proteome profiling by SEC‐SWATH‐MS
Source: Mol Syst Biol. 2019 Jan 14;15(1):e8438. doi: 10.15252/msb.20188438 (PMC6346213; doi:10.15252/msb.20188438)
Supplement: Supplementary file 8 — Dataset EV7 [file MSB-15-e8438-s008.zip › feature_plots_string/Q14690.pdf]

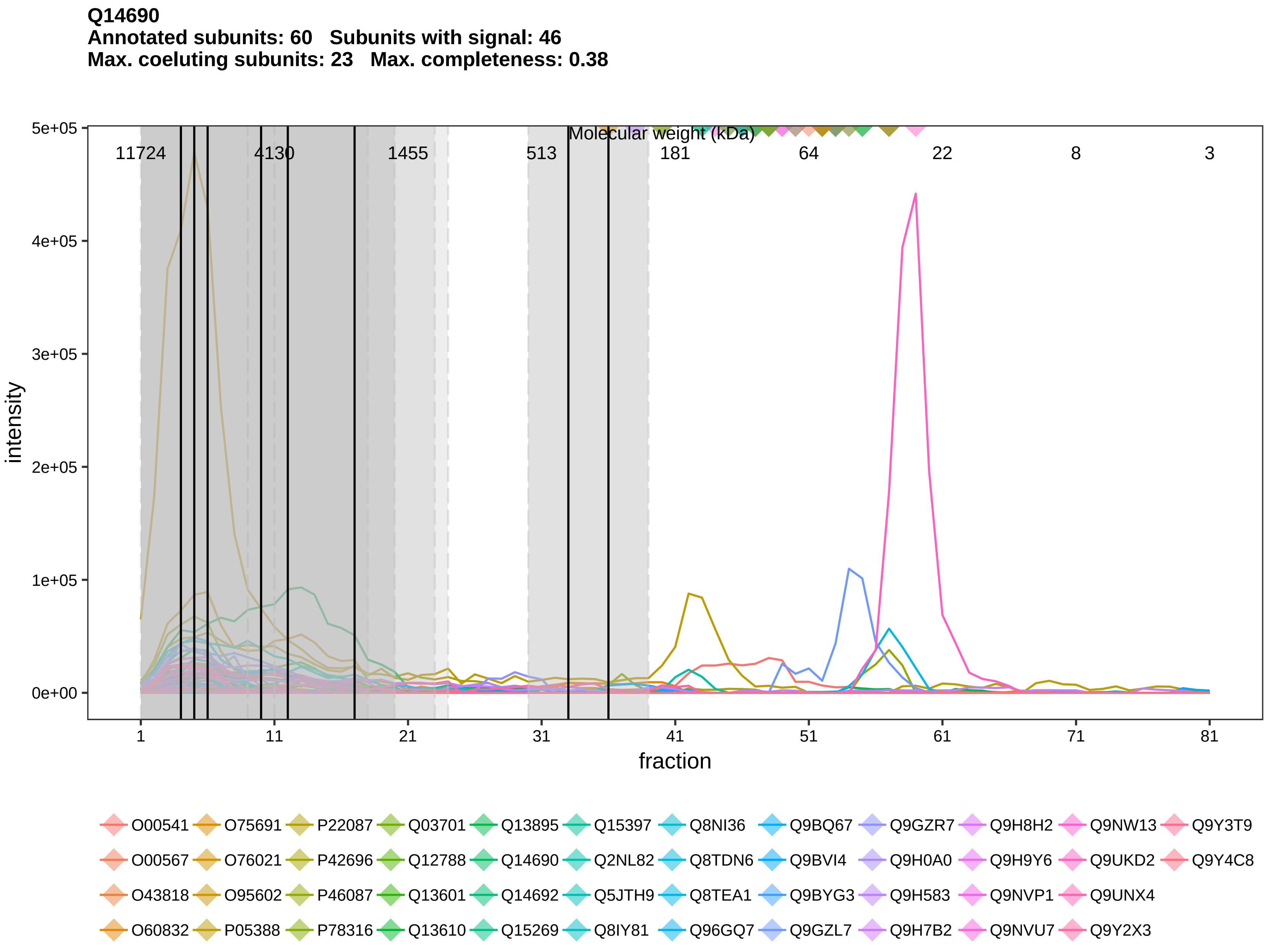
Q14690
Annotated subunits: 60 Subunits with signal: 46
Max. coeluting subunits: 23 Max. completeness: 0.38
Molecular weight (kDa)
11724
4130
1455
513
181
64
22
8
3
intensity
5e+05
4e+05
3e+05
2e+05
1e+05
0e+00
fraction
1
11
21
31
41
51
61
71
81
O00541
O75691
P22087
Q03701
Q13895
Q15397
Q8NI36
Q9BQ67
Q9GZR7
Q9H8H2
Q9NW13
Q9Y3T9
O00567
O76021
P42696
Q12788
Q14690
Q2NL82
Q8TDN6
Q9BVI4
Q9H0A0
Q9H9Y6
Q9UKD2
Q9Y4C8
O43818
O95602
P46087
Q13601
Q14692
Q5JTH9
Q8TEA1
Q9BYG3
Q9H583
Q9NVP1
Q9UNX4
O60832
P05388
P78316
Q13610
Q15269
Q8IY81
Q96GQ7
Q9GZL7
Q9H7B2
Q9NVU7
Q9Y2X3
